# Relationship between postprandial changes in cardiac left ventricular function, glucose and insulin concentrations, gastric emptying, and satiety in healthy subjects

**DOI:** 10.1186/1475-2891-10-26

**Published:** 2011-03-23

**Authors:** Joanna Hlebowicz, Sandra Lindstedt, Ola Björgell, Magnus Dencker

**Affiliations:** 1 Department of Medicine/Cardiology, Lund University, Skåne University Hospital, Malmö, Sweden; 2 Department of Cardiothoracic Surgery, Lund University, Skåne University Hospital, Lund, Sweden; 3 Department of Radiology, Lund University, Skåne University Hospital, Malmö, Malmö, Sweden; 4 Department of Clinical Physiology and Nuclear Medicine, Lund University, Skåne University Hospital, Malmö, Sweden

## Abstract

**Background:**

The digestion of food is known to alter the hemodynamics of the body significantly. The purpose of this study was to study the postprandial changes in stroke volume (SV), cardiac output (CO) and left ventricular (LV) longitudinal systolic and diastolic functions measured with tissue Doppler imaging, in relation to gastric emptying rate (GER), satiety, and glucose and insulin concentrations in healthy subjects.

**Methods:**

Twenty-three healthy subjects were included in this study. The fasting and postprandial changes at 30 min and 110 min in CO, heart rate (HR) and blood pressure were measured. Moreover, tissue Doppler imaging systolic (S'), early (E') and late (A') mitral annular diastolic velocities were measured in the septal (s) and lateral (l) walls. Glucose and insulin concentrations, and satiety were measured before and 15, 30, 45, 60, 90, and 120 min after the start of the meal. The GER was calculated as the percentage change in the antral cross-sectional area 15-90 min after ingestion of the meal.

**Results:**

This study show that both CO, systolic longitudinal ventricular velocity of the septum (S's) and lateral wall (S'l), the early diastolic longitudinal ventricular velocity of the lateral wall (E'l), the late diastolic longitudinal ventricular velocity of the septum (A's) and lateral wall (A'l) increase significantly, and were concomitant with increased satiety, antral area, glucose and insulin levels. The CO, HR and SV at 30 min were significantly higher, and the diastolic blood pressure was significantly lower, than the fasting. The satiety was correlated to HR and diastolic blood pressure. The insulin level was correlated to HR.

**Conclusions:**

This study shows that postprandial CO, HR, SV and LV longitudinal systolic and diastolic functions increase concomitantly with increased satiety, antral area, and glucose and insulin levels. Therefore, patients should not eat prior to, or during, cardiac evaluation as the effects of a meal may affect the results and their interpretation.

**Trial Registration:**

ClinicalTrials.gov: NCT01027507

## Background

The digestion of food is known to alter the hemodynamics of the body significantly. Following a meal, the blood flow to the gastrointestinal organs increases, affecting the heart rate (HR), blood pressure, and cardiac output (CO). The mechanisms causing the change in heart function after a meal are not known. These postprandial cardiovascular changes have been shown to resemble the effects of vasodilator drugs [1,2].

The left ventricular (LV) stroke volume (SV) and CO are usually assessed by echocardiography to quantify the total LV function. However, the heart undergoes cyclic mechanical changes in multiple dimensions that result in the ejection of blood. Tissue Doppler imaging (TDI) may be used to quantify different aspects of mechanical myocardial activity. TDI is used to assess both regional and the global ventricular function in systole and diastole, by measuring the tissue velocity at specific locations in the heart. Tissue velocity is determined by the rate at which specific parts of the myocardium move towards or away from the transducer. TDI has been validated and may have a potential role in clinical applications such as the evaluation of myocardial ischemia (at rest and with stress echocardiography) and altered global and regional systolic and diastolic function in cardiomyopathies [3-11].

The systolic longitudinal velocity (S') of a myocardial segment measured with TDI reflects the systolic motion of the LV in the apical direction, which is an important component of systolic function [12], and is correlated with the LV ejection fraction [8]. S' may be used to diagnose impaired LV systolic function [8,12,13]. Early (E') and late (A') mitral annular diastolic velocities are directed in the opposite direction of S' and have been used to diagnose diastolic heart failure [14], or early hypertrophic cardiomyopathy [15]. Moreover, mitral annular velocities, measured with TDI, have been shown to predict mortality and cardiovascular events [16,17].

The effect of the digestion of a meal on left ventricular function has not previously been studied in terms of longitudinal velocity measured with TDI. This study was therefore designed to determine whether there are postprandial changes in LV function that can be measured with TDI, and if these changes can be related to changes in glucose and insulin levels, antral area or satiety in healthy subjects.

## Methods

Twenty-three healthy subjects [11 male, 12 female; [mean ± SEM] age: 26 ± 0.2 y [range: 18-33 y]; body mass index: 21.8 ± 0.1 kg/m^2 ^[range: 17.0-25.9 kg/m^2^] without symptoms or a history of gastrointestinal disease, abdominal surgery or diabetes mellitus, were included in this observational study. The mean waist:hip ratio for the women was 0.74 ± 0.02 and for the men 0.87 ± 0.01. The subjects had no connective tissue disease or cerebrovascular or endocrine disease, and none was taking any medication, except four women who were taking oral contraceptives. All subjects were in sinus rhythm. Three men were snuff users, and one was a smoker. Two women were snuff users. All subjects were recruited from the population of southern Sweden.

The subjects were examined between 7.30 and 11.00 am after an 8-h fast. Smoking and snuff-taking were prohibited 8 h prior to and during the test. The fasting blood glucose concentration of each subject was checked on the day of the examination to ensure that it was normal. If the subjects reported gastrointestinal symptoms (diarrhea or constipation) on the day of the study, the examination was postponed. The test meal consisted of 300 g rice pudding (Goda Gröten Risgrynsgröt; Lantmännen AXA, Järna, Sweden). The total caloric value of the meal was 330 kcal: 10% from protein (9 g), 58% from carbohydrates (48 g) and 32% from fat (12 g). The meal was ingested within 5 min.

The gastric emptying rate (GER) was estimated using an ultrasound method described in detail previously [18]. The sonographic examination was performed with a 3.5 MHz abdominal transducer (Acuson Sequioa 512, Mountain View, CA), and an imaging system (Siemens Elegra, Siemens Medical Solutions, Mountain View, CA). Measurements of the gastric antrum were performed by a single observer. The abdominal aorta and the left lobe of the liver were used as internal landmarks in each measurement of the gastric antrum. The subjects were examined in the supine position, and not allowed to sit up between examinations. Measurements were made 15 and 90 min after the meal had been consumed, and the GER was expressed as the percentage change in the antral cross-sectional area between these two measurements. At each examination, the longitudinal and anteroposterior diameters were measured three times, and the mean values were used to calculate the cross-sectional area of the gastric antrum. The GER (%) was calculated using the following equation:

Transthoracic echocardiography examinations were performed with a Sonos 5500 ultrasound system (Philips, Andover, MA, USA) in the left lateral position, after 15 minutes' rest. On the day of the study, an initial examination was performed to rule out any cardiac dysfunction. A single observer performed all echocardiography measurements three times on separate cardiac cycles, and the mean values were used in the analyses. TDI of the septal (s) and lateral (l) mitral annulus was obtained from the apical four-chamber view, in accordance with current guidelines [19]. Peak systolic (S'), early (E') and late (A') mitral annular diastolic velocities were measured. The LV SV was measured, and the LV CO was calculated according to current guidelines [20,21]. All TDI measurements were performed under respiratory arrest after an end-expiration. The blood pressure and echocardiogram were measured before the meal (0 min) and 30, and 110 min after the start of the meal.

Venous blood samples were taken before and 15, 30, 45, 60, 90, and 120 min after the start of the meal to measure blood glucose and plasma insulin levels. Blood glucose concentrations were measured with the HemoCue Glucose system (HemoCue AB, Ängelholm, Sweden). The precision of the HemoCue Glucose system was better than 0.3 SD from 0 mmol/L to 22.2 mmol/L. Insulin concentrations were measured using an immunoassay with an alkaline phosphatase conjugate (Access Ultrasensitive Insulin, Beckman-Coulter AB, Bromma, Sweden). The sensitivity of the insulin immunoassay was 0.03 mUnit/L (mU/L), and the intra-assay coefficient of variation was below 10% from 0.03 mU min/L to 300 mU/L.

A validated satiety scoring scale was used according to the method of Hauber et al., based on a scoring system from -10 (extreme hunger) to +10 (extreme satiety) [22]. Satiety scores were estimated before the meal (0 min) and 15, 30, 45, 60, 90 and 120 min after the start of the meal.

All subjects gave their written informed consent. The study was approved by the Ethics Committee of Lund University, and performed according to the Helsinki Declaration. The trial is registered in the US National Library of Medicine with the trial registration number NCT01027507.

Results are given as mean values and the SEM unless otherwise stated. The areas under the curves (AUCs) were calculated for blood glucose and insulin concentrations, satiety, CO, HR, SV, systolic and diastolic blood pressure, S's, S'l, E's, E'l, A's, and A'l in each subject using GraphPad Prism software (version 4; GraphPad, San Diego, CA). All other statistical calculations were performed in SPSS for Windows version 14.0, 2005 (SPSS Inc., Chicago IL, USA). The changes in levels of blood glucose, plasma insulin and satiety were calculated as the difference between levels before the meal (fasting value) and 30 and 120 min after the start of the meal. The changes in HR, SV, CO, systolic and diastolic blood pressure, S's, S'l, E's, E'l, A's, and A'l were calculated as the difference between levels before the meal (fasting value) and 30 and 110 min after the start of the meal. To determine whether the meal affected a given parameter, the baseline value was compared with the 30 min and 120 min postprandial value using the Wilcoxon t-test. The postprandial values at 30 min and 120 min were also compared using the Wilcoxon t-test. Possible correlations between CO, HR, SV, systolic blood pressure, diastolic blood pressure, S's, S'l, E's, E'l, A's, A'l, blood glucose level, plasma insulin level, GER, and satiety were analyzed with Pearson's correlation. Values of *P *< 0.05 were considered significant.

## Results

### Postprandial glucose and insulin responses

**Figure 1:**
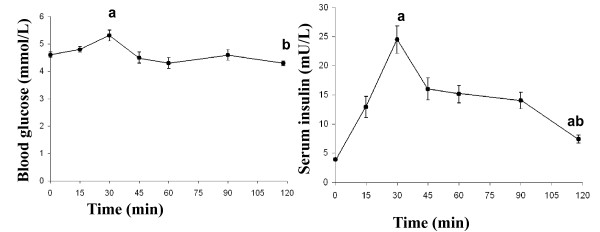
**The mean (± SEM) blood glucose (left) and serum insulin (right) concentrations in 23 healthy subjects after the ingestion of a meal consisting of rice pudding**. 'a' denotes a significantly different value in the postprandial phase compared to fasting, according to the Wilcoxon t-test (*P *< 0.05). 'b' denotes a significantly different value in the postprandial phase at 30 min compared to 120 min, according to the Wilcoxon t-test (*P *< 0.000).

The glucose level at 30 min was significantly higher than the baseline value (*P *= 0.003), and the value at 120 min (*P *= 0.000) (Figure 1). The insulin levels at both 30 min and 120 min were significantly higher than the baseline value (*P *= 0.000). The insulin level at 30 min was significantly higher than at 120 min (*P *= 0.000) (Figure 1).

### Gastric emptying rate

The mean value of the antral cross-sectional area was significantly larger 15 min after the end of the meal than 90 min after the meal (667 ± 48 mm^2 ^vs. 384 ± 41 mm^2^, P = 0.000). The mean value of the GER after the meal was estimated to be 43 ± 4%.

### Satiety

**Figure 2:**
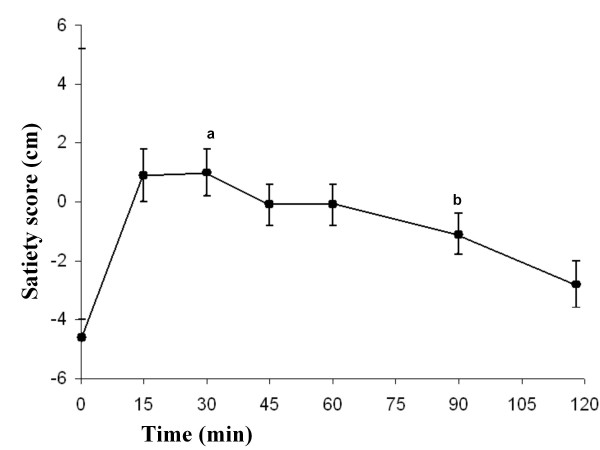
**The mean (± SEM) satiety scores in 23 healthy subjects after the ingestion of a meal consisting of rice pudding**. 'a' denotes a significantly different value in the postprandial phase compared to fasting, according to the Wilcoxon t-test (*P *< 0.000). 'b' denotes a significantly different value in the postprandial phase at 30 min compared to 120 min, according to the Wilcoxon t-test (*P *< 0.000).

The satiety 30 min after the meal was significantly higher than the baseline value (*P *= 0.000). The satiety 120 min after the meal was significantly lower than at 30 min (*P *= 0.000) (Figure 2).

### Cardiovascular parameters

**Figure 3:**
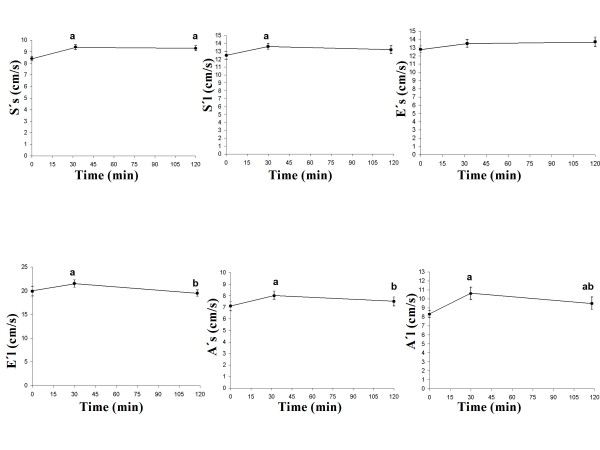
**Mean values (± SEM) of S's, S'l, E's, E'l, A's, and A'l in 23 healthy subjects after the ingestion of a meal consisting of rice pudding**. 'a' denotes a significantly different value in the postprandial phase compared to fasting, according to the Wilcoxon t-test (*P *< 0.05). 'b' denotes a significantly different value in the postprandial phase at 30 min compared to 120 min, according to the Wilcoxon t-test (*P *< 0.05).

**Figure 4:**
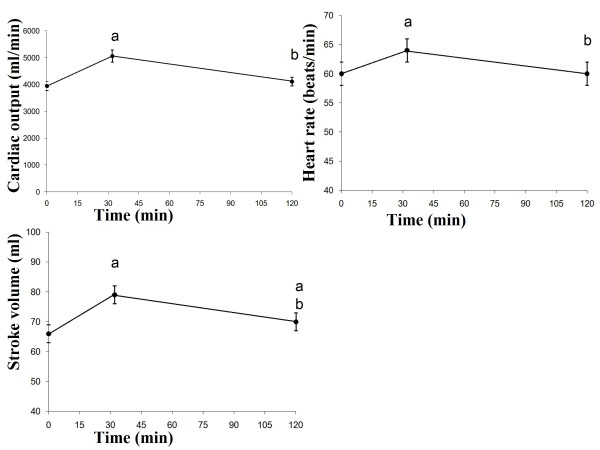
**Mean values (± SEM) of cardiac output, heart rate and stroke volume in 23 healthy subjects after the ingestion of a meal consisting of rice pudding**. 'a' denotes a significantly different value in the postprandial phase compared to fasting, according to the Wilcoxon t-test (*P *< 0.05). 'b' denotes a significantly different value in the postprandial phase at 30 min compared to 120 min, according to the Wilcoxon t-test (*P *< 0.05).

**Figure 5:**
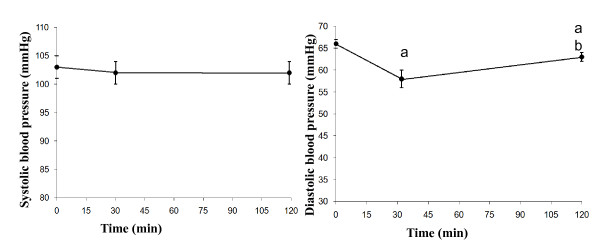
**Mean values (± SEM) of systolic and diastolic blood pressure in 23 healthy subjects after the ingestion of a meal consisting of rice pudding**. 'a' denotes a significantly different value in the postprandial phase compared to fasting, according to the Wilcoxon t-test (*P *< 0.05). 'b' denotes a significantly different value in the postprandial phase at 30 min compared to 120 min, according to the Wilcoxon t-test (*P *< 0.05).

The S'l at 30 min was significantly higher than the baseline value (*P *= 0.016). The E'l at 30 min was significantly higher than at baseline (*P *= 0.038), and at 110 min (*P *= 0.003). The A's at 30 min was significantly higher than at baseline (*P *= 0.001), and at 110 min (*P *= 0.006). The A'l at baseline was significantly lower than at 30 min (*P *= 0.000), and at 110 min (*P *= 0.005). The A'l at 30 min was significantly higher than at 110 min (*P *= 0.041) (Figure 3). The CO at 30 min was significantly higher than the fasting and at 110 min (both *P *= 0.000). The HR at 30 min was significantly higher than fasting and at 110 min (*P *= 0.009 and *P *= 0.002, respectively). The SV at 30 min was significantly higher than fasting and at 110 min (both *P *= 0.000). The SV at 30 min was significantly higher than at 110 min (*P *= 0.003) (Figure 4). The systolic blood pressure at fasting, at 30 min and at 110 min was not significantly different. The diastolic blood pressure at 30 min was significantly lower than the fasting and at 110 min (*P *= 0.000, *P *= 0.026, respectively). The diastolic blood pressure at 30 min was significantly lower than at 110 min (*P *= 0.001) (Figure 5).

## Correlations

At 30 min there was a significant correlation between E'l and glucose level (*P = *0.036, r = -0.44) and antrum area at 15 min (*P *= 0.000, r = -0.67). At 30 min there was also a significant correlation between the E's and satiety (*P *= 0.028, r = -0.45). A significant correlation was found between E'l at 110 min and antral area at 90 min (*P *= 0.020, r = -0.48), and between E's at 110 min and GER (*P *= 0.042, r = -0.43). There was a significant correlation between the change in A's at 30 min and the change in satiety (*P *= 0.008, r = -0.54). There was also a significant correlation between the change in A's at 110 min and the change in insulin level (*P *= 0.029, r = -0.47). A significant correlation was found between the change in E's at 110 min and GER (*P *= 0.006, r = -0.55). There was significant correlation between the glucose AUC at 30 min and the AUC for E'l at 30 min (*P *= 0.029, r = -0.46). A significant correlation was also found between the satiety AUC at 120 min and the AUC for S'l at 110 min (*P *= 0.049, r = 0.41). There was significant correlation between the satiety AUC at 30 min and the AUC for HR at 30 min and the AUC for the diastolic blood pressure at 30 min (*P *= 0.028, r = -0.46; *P *= 0.021, r = -0.48, respectively). A significant correlation was found between the insulin AUC at 120 min and HR at 110 min (*P *= 0.023, r = -0.48).

## Discussion

The purpose of this study was to measure the postprandial changes in LV longitudinal, systolic and diastolic functions, using TDI, and to investigate possible correlations to GER, satiety, and glucose and insulin concentrations in healthy subjects. Our hypothesis was that an intake of food would change LV function, and that this change would be related to postprandial changes in glucose and insulin levels, the antral area of the stomach or satiety. In this study, postprandial values of S's, S'l, E'l, A's, and A'l, increased significantly and concomitantly with increases in glucose and insulin levels, antral area, and satiety. LV contractions result in shortening of both the short and long axes of the left ventricle. The longitudinal movement of the left ventricle has been shown to be correlated with the LVEF [12]. Since mechanical myocardial activity and regional functions result in the ejection of blood, it is not surprising that the longitudinal systolic function, as measured as tissue velocity, also increases postprandially. Previous studies have shown that the ingestion of a meal increases the CO, HR, and SV [23-25] and decreases diastolic blood pressure [26]. This is in concordance with the results in present investigation which showed that postprandial CO, HR and SV increased. Diastolic blood pressure decreased and no change was observed for systolic blood pressure. The satiety was correlated to HR and diastolic blood pressure. The insulin level was also correlated to postprandial changes in HR.

The autonomic innervation of the heart and stomach is divided into the parasympathetic and sympathetic systems. Sympathetic stimulation increases heart rate (positive chronotropy), inotropy and conduction velocity (positive dromotropy), whereas parasympathetic stimulation of the heart has the opposite effects. Changes in HR variability and blood pressure may reflect changes in the balance between the sympathetic and parasympathetic nervous systems. Postprandial hemodynamic changes could be mediated by the innervation of the heart. The vagus nerve mediates the adaptive relaxation of the proximal stomach, the fundic-antral co-ordination (by controlled delivery of the food from the fundus into the antrum), and the peristaltic contractions of the distal stomach after a meal [27]. The endocrine system, the autonomic nervous system and the antral area have been shown to be important in the control of satiety and hunger [28-33]. In our study, the ingestion of food led to a decrease in the diastolic blood pressure, without affecting the systolic blood pressure. The postprandial satiety was correlated to the HR and diastolic blood pressure. Previous studies have shown that gastric distension influences blood pressure, probably due to activation of the gastrovascular reflex in patients with autonomic failure [34-36], and in older healthy subjects [37]. However, the systolic blood pressure was not affected in healthy adolescents [35].

Postprandial changes in heart activity may be influenced by changes in glucose and hormonal signals. It is known that insulin has hemodynamic effects, such as positive chronotropic and inotropic effects on the heart [38]. In this study the postprandial insulin level was correlated to the HR. To the best of our knowledge, the relations between increasing antral area, gastric emptying rate, and satiety on postprandial changes in LV function measured with TDI, have not been studied previously. In this study, the antral cross-sectional area at 15 and 90 min was not correlated to the postprandial changes in E'l, while the GER was correlated to E's. This study shows that postprandial changes in glucose levels are correlated to E'l, and that the insulin level is correlated to A's. Intravenous insulin administration has previously been reported to increase the CO before any significant decrease in glucose levels was seen in healthy subjects [39,40]. The endocrine system, the autonomic nervous system and the antral area have been shown to be important in the control of satiety and hunger [28-33]. We found that satiety was correlated to E's, S'l and A's. Therefore, it appears that postprandial glucose and insulin levels, and antral distention may play a role in the postprandial hemodynamic response of the heart. It is reasonable to suggest that the changes observed are partly related to the increased heart work and subsequent changes in loading conditions. The exact mechanisms for these relationships remains, however, undermined and represent an area for future investigations.

A limitation of the present study was the fact that it was not possible to perform the echocardiography or gastric empting examinations at the same time, or blinded to the state of food intake. However, both measurements were performed by a single observer thereby avoiding intra-observer variability. The echocardiography examinations were stored digitally and analyzed later in random order in an attempt to avoid bias.

## Conclusions

This study shows that both postprandial LV global and longitudinal function increase concomitantly with increased satiety, antral area, and glucose and insulin levels. Patients should therefore not eat prior to or during cardiac evaluation because the effects of a meal may affect the results and their interpretation.

## Abbreviations

A's: late longitudinal ventricular velocity of the septum wall; A'l: late longitudinal ventricular velocity of the lateral wall; CO: cardiac output; E's: early longitudinal ventricular velocity of the septum wall; E'l: early longitudinal ventricular velocity of the lateral wall; GER: gastric emptying rate; HR: heart rate; LV: left ventricular; S's: systolic longitudinal ventricular velocity of the septum wall; S'l: systolic longitudinal ventricular velocity of the lateral wall; SV: stroke volume; TDI: Tissue Doppler imaging

## Competing interests

The authors declare that they have no competing interests.

## Authors' contributions

The authors' contributions were as follows: JH and MD contributed to the design of the study; JH was responsible for recruiting the subjects and carried out the practical aspects of the study. OB performed the ultrasound examinations; MD performed the echocardiographic examinations; JH, SL, and MD conducted the statistical calculations; JH, SL, and MD created the graphs. JH and MD wrote the first draft of the manuscript and SL and OB made critical revisions of the manuscript. All authors read and approved the final manuscript. None of the authors had any personal or financial conflict of interest.

## References

[B1] Waaler BA Hisdal J Eriksen M Circulatory responses to a meal in patients with a newly transplanted heart Acta Physiol Scand 2002 174 101 108 10.1046/j.1365-201X.2002.00934.x 11860371

[B2] Packer M Medina N Yushak M Hemodynamic changes mimicking a vasodilator drug response in the absence of drug therapy after right heart catheterization in patients with chronic heart failure Circulation 1985 71 761 766 3971544 10.1161/01.cir.71.4.761

[B3] Sanderson JE Wang M Yu CM Tissue Doppler imaging for predicting outcome in patients with cardiovascular disease Curr Opin Cardiol 2004 19 5 458 463 10.1097/01.hco.0000133110.58863.52 15316453

[B4] Isaaz K Thompson A Ethevenot G Cloez JL Brembilla B Pernot C Doppler echocardiographic measurement of low velocity motion of the left ventricular posterior wall Am J Cardiol 1989 64 66 75 10.1016/0002-9149(89)90655-3 2741815

[B5] De Boeck BW Cramer MJ Oh JK van der Aa RP Jaarsma W Spectral pulsed tissue Doppler imaging in diastole: a tool to increase our insight in and assessment of diastolic relaxation of the left ventricle Am Heart J 2003 146 411 419 10.1016/S0002-8703(03)00322-3 12947357

[B6] Hatle L Sutherland GR Regional myocardial function - a new approach Eur Heart J 2000 21 1337 1357 10.1053/euhj.2000.2251 10952824

[B7] Abraham TP Dimaano VL Liang HY Role of tissue Doppler and strain echocardiography in current clinical practice Circulation 2007 116 2597 2609 10.1161/CIRCULATIONAHA.106.647172 18040039

[B8] Yu CM Sanderson JE Marwick TH Oh JK Tissue Doppler imaging a new prognosticator for cardiovascular diseases J Am Coll Cardiol 2007 49 1903 1914 10.1016/j.jacc.2007.01.078 17498573

[B9] Citro R Bossone E Kuersten B Gregorio G Salustri A Tissue Doppler and strain imaging: anything left in the echo-lab? Cardiovasc Ultrasound 2008 6 54 10.1186/1476-7120-6-54 18973677 PMC2583989

[B10] Van de Veire NR De Sutter J Bax JJ Roelandt JR Technological advances in tissue Doppler imaging echocardiography Heart 2008 94 1065 1074 10.1136/hrt.2007.120758 18230638

[B11] Ng AC Thomas L Leung DY Tissue Doppler echocardiography Minerva Cardioangiol 2010 58 357 378 20485241

[B12] Henein MY Gibson DG Long axis function in disease Heart 1999 81 229 231 10026340 10.1136/hrt.81.3.229 PMC1728969

[B13] Sanderson JE Heart failure with a normal ejection fraction Heart 2007 93 155 158 10.1136/hrt.2005.074187 16387829 PMC1861394

[B14] Fang ZY Leano R Marwick TH Relationship between longitudinal and radial contractility in subclinical diabetic heart disease Clin Sci (Lond) 2004 106 53 60 10.1042/CS20030153 12924982

[B15] Nagueh SF Bachinski LL Meyer D Hill R Zoghbi WA Tam JW Quiñones MA Roberts R Marian AJ Tissue Doppler imaging consistently detects myocardial abnormalities in patients with hypertrophic cardiomyopathy and provides a novel means for an early diagnosis before and independently of hypertrophy Circulation 2001 104 128 130 11447072 10.1161/01.cir.104.2.128 PMC2900859

[B16] Wang M Yip GW Wang AY Zhang Y Ho PY Tse MK Lam PK Sanderson JE Peak early diastolic mitral annulus velocity by tissue Doppler imaging adds independent and incremental prognostic value J Am Coll Cardiol 2003 41 820 826 10.1016/S0735-1097(02)02921-2 12628728

[B17] Wang M Yip GW Wang AY Zhang Y Ho PY Tse MK Yu CM Sanderson JE Tissue Doppler imaging provides incremental prognostic value in patients with systemic hypertension and left ventricular hypertrophy J Hypertens 2005 23 183 191 10.1097/00004872-200501000-00029 15643141

[B18] Darwiche G Almér LO Björgell O Cederholm C Nilsson P Measurement of gastric emptying by standardized real-time ultrasonography in healthy subjects and diabetic patients J Ultrasound Med 1999 18 10 673 682 10511299 10.7863/jum.1999.18.10.673

[B19] Nagueh SF Appleton CP Gillebert TC Marino PN Oh JK Smiseth OA Waggoner AD Flachskampf FA Pellikka PA Evangelista A Recommendations for the evaluation of left ventricular diastolic function by echocardiography J Am Soc Echocardiogr 2009 22 107 133 10.1016/j.echo.2008.11.023 19187853

[B20] Quiñones MA Otto CM Stoddard M Waggoner A Zoghbi WA Recommendations for quantification of Doppler echocardiography: a report from the Doppler Quantification Task Force of the Nomenclature and Standards Committee of the American Society of Echocardiography J Am Soc Echocardiogr 2002 15 167 184 11836492 10.1067/mje.2002.120202

[B21] Lang RM Bierig M Devereux RB Flachskampf FA Foster E Pellikka PA Picard MH Roman MJ Seward J Shanewise JS Solomon SD Spencer KT Sutton MS Stewart WJ Chamber Quantification Writing Group; American Society of Echocardiography's Guidelines and Standards Committee;European Association of Echocardiography Recommendations for chamber quantification: a report from the American Society of Echocardiography's Guidelines and Standards Committee and the Chamber Quantification Writing Group, developed in conjunction with the European Association of Echocardiography, a branch of the European Society of Cardiology J Am Soc Echocardiogr 2005 18 1440 1463 10.1016/j.echo.2005.10.005 16376782

[B22] Haber GB Heaton KW Murphy D Burroughs LF Depletion and disruption of dietary fibre effects on satiety, plasma glucose, and serum-insulin Lancet 1977 2 8040 679 682 10.1016/S0140-6736(77)90494-9 71495

[B23] Waaler BA Eriksen M Toska K The effect of meal size on postprandial increase in cardiac output Acta Physiol Scand 1991 142 1 33 39 10.1111/j.1748-1716.1991.tb09125.x 1877363

[B24] Waaler BA Eriksen M Post-prandial cardiovascular responses in man after ingestion of carbohydrate, protein or fat Acta Physiol Scand 1992 146 3 321 327 10.1111/j.1748-1716.1992.tb09426.x 1481689

[B25] Fagan TC Sawyer PR Gourley LA Lee JT Gaffney TE Postprandial alterations in hemodynamics and blood pressure in normal subjects Am J Cardiol 1986 58 636 641 10.1016/0002-9149(86)90291-2 3751935

[B26] Waaler BA Hisdal J Eriksen M Circulatory responses to a meal in patients with a newly transplanted heart Acta Physiol Scand 2002 174 101 108 10.1046/j.1365-201X.2002.00934.x 11860371

[B27] Sheiner HJ Quinlan MF Thompson IJ Gastric motility and emptying in normal and post-vagotomy subjects Gut 1980 21 9 753 759 10.1136/gut.21.9.753 7429340 PMC1419535

[B28] Jones KL Doran SM Hveem K Bartholomeusz FD Morley JE Sun WM Chatterton BE Horowitz M Relation between postprandial satiation and antral area in normal subject Am J Clin Nutr 1997 66 127 132 9209180 10.1093/ajcn/66.1.127

[B29] Santangelo A Perracchi M Conte D Fraquelli M Porrini Physical state of meal gastric emptying, cholecystokinin release and satiety Br J Nutr 1998 80 521 527 10211050 10.1017/s0007114598001615

[B30] Bergmann JF Chassany O Petit A Triki R Caulin C Segrestaa JM Correlation between echographic gastric emptying and appetite: influence of psyllium Gut 1992 33 1042 1043 10.1136/gut.33.8.1042 1398229 PMC1379439

[B31] Sturm K Parker B Wishart J Feinle-Bisset C Jones KL Chapman I Horowitz M Energy intake and appetite are related to antral area in healthy young and older subjects Am J Clin Nutr 2004 80 656 667 15321806 10.1093/ajcn/80.3.656

[B32] Hveem K Jones KL Chatterton BE Horowitz M Scintigraphic measurment of gastric empying and ultrasonographic assessment of antral area: relation to appetite Gut 1996 8 816 821 10.1136/gut.38.6.816 PMC1383185 8984016

[B33] Näslund E Hellström PM Appetite signaling: from gut peptides and enteric nerves to brain Physol Behav 2007 92 1-2 256 262 10.1016/j.physbeh.2007.05.017 17582445

[B34] Cariga P Mathias CJ Haemodynamics of the pressor effect of oral water in human sympathetic denervation due to autonomic failure Clin Sci (Lond) 2001 101 3 313 319 10.1042/CS20000253 11524049

[B35] Jordan J Shannon JR Grogan E Biaggioni I Robertson D A potent pressor response elicited by drinking water Lancet 1999 353 9154 723 10.1016/S0140-6736(99)99015-3 10073520

[B36] Jordan J Shannon JR Black BK Ali Y Farley M Costa F Diedrich A Robertson RM Biaggioni I Robertson D The pressor response to water drinking in humans: a sympathetic reflex? Circulation 101 5 504 509 202 10662747 10.1161/01.cir.101.5.504

[B37] Gentilcore D Meyer JH Rayner CK Horowitz M Jones C Gastric distension attenuates the hypotensive effect of intraduodenal glucose in healthy older subjects Am J Physiol Regul Integr Comp Physiol 2008 295 2 R472 477 10.1152/ajpregu.00108.2008 18495838

[B38] Baron AD Hemodynamic actions of insulin Am J Physiol 1994 267 E187 202 8074198 10.1152/ajpendo.1994.267.2.E187

[B39] Fisher BM Gillen G Dargie HJ Inglis GC Frier BM The effects of insulin induced hypoglycemia on cardiovascular function in normal man: studies using radionuclide ventriculography Diabetologia 1987 30 841 845 10.1007/BF00275756 3328719

[B40] Baron AD Brechtel G Insulin differentially regulates systemic and skeletal muscle vascular resistance Am J Physiol 1993 265 E61 67 8338155 10.1152/ajpendo.1993.265.1.E61

